# Novel, Fully Characterised Bovine Taste Bud Cells of Fungiform Papillae

**DOI:** 10.3390/cells10092285

**Published:** 2021-09-02

**Authors:** Habtom Ftuwi, Rheinallt Parri, Afzal R. Mohammed

**Affiliations:** Aston Pharmacy School, Aston University, Birmingham B4 7ET, UK; ftuwih@aston.ac.uk (H.F.); h.r.parri@aston.ac.uk (R.P.)

**Keywords:** taste bud cells, taste cell characterization, PCR

## Abstract

Current understanding of functional characteristics and biochemical pathways in taste bud cells have been hindered due the lack of long-term cultured cells. To address this, we developed a holistic approach to fully characterise long term cultured bovine taste bud cells (BTBCs). Initially, cultured BTBCs were characterised using RT-PCR gene expression profiling, immunocytochemistry, flowcytometry and calcium imaging, that confirmed the cells were mature TBCs that express taste receptor genes, taste specific protein markers and capable of responding to taste stimuli, i.e., denatonium (2 mM) and quinine (462.30 μM). Gene expression analysis of forty-two genes implicated in taste transduction pathway (map04742) using custom-made RT-qPCR array revealed high and low expressed genes in BTBCs. Preliminary datamining and bioinformatics demonstrated that the bovine α-gustducin, gustatory G-protein, have higher sequence similarity to the human orthologue compared to rodents. Therefore, results from this work will replace animal experimentation and provide surrogate cell-based throughput system to study human taste transduction.

## 1. Introduction

Sense of taste has the most important role in food choice and eating habits. Food substances (or taste chemicals) activate specialised sensory cells of the taste bud cells (TBCs) which convey taste signals to the brain. This signal is then decoded in the brain to distinguish the taste type and intensity; and this taste perception has a key role in guiding nutrition consumption, avoiding toxic substances and maintenance of a healthy diet [1]. Taste bud cells (TBC) are specialised epithelial cells in the oral cavity; they are grouped as neuroepithelial cells given their sensory role and ability to communicate through release of several neurotransmitters (e.g., ATP, serotonin, glutamate and GABA). Taste bud cells (TBC) are broadly classified in to four subtypes: basal, Type I, Type II (taste receptor cells) and Type III cells.

Current understanding of lineage, functional characteristics and biochemical pathways of TBCs have been hindered due the lack of long-term cultured cells. Thus, studies were performed in explant taste buds, slices of lingual tissue and intact taste buds [2,3,4,5,6] or heterogeneous expressing systems using HEK293T cells [7,8,9,10].

Long term TBCs in vitro culture and maintenance had been a significant challenge as TBCs degenerate and disappear following denervation [11]. Kishi et al. attempted an in vitro culture of TBCs, but cells were only viable for three days [2].

However, work from Ozdener group laid the foundation for optimisation and evaluation of long-term cultures from rat fungiform papillae [12] and then from human fungiform papillae [13]. The current study aims to develop fully characterised long-term cell culture lines from bovine taste bud cells (BTBC). The fungiform taste buds on the bovine tongue are evident structure, i.e., popped structure; and this enables to precisely isolate the taste buds as well as harvest significantly larger yield. A holistic approach including morphology characterisation, gene expression analyses, determination of cell sub types and functional characterisation was studied to confirm phenotypic, genotypic and functional properties of BTBCs. Furthermore, gene expression analysis using custom made RT-qPCR array and bioinformatics were employed to characterise BTBCs.

## 2. Materials and Methods

**Figure 1:**
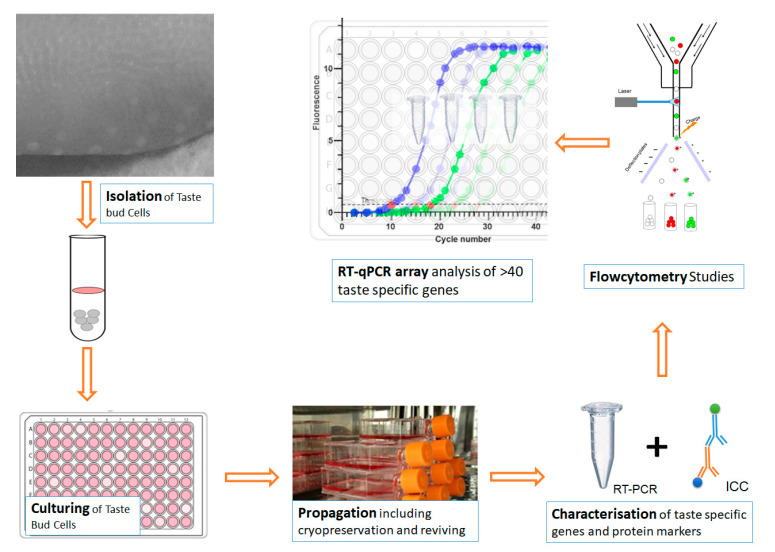
A schematic diagram showing the process of BTBCs isolation, culturing, characterisation using RT-PCR and immunocytochemistry followed by flow cytometry studies and RT-qPCR array analysis of taste specific genes.

The process of BTBCs isolation, culturing, characterisation of cultured BTBCs using RT-PCR and immunocytochemistry followed by flow cytometry studies and RT-qPCR array analysis of taste specific genes and coupled with preliminary bioinformatics studies were investigated in this study (Figure 1).

### 2.1. Isolation and Culturing of BTBCs

The protocol for isolation and culturing bovine taste bud cells (BTBCs) was adapted from Ozdener et al. [12]. Freshly obtained bovine tongues were obtained from local abattoir (C. H. Rowley LTD) in temperature-controlled conditions. Taste bud isolation was performed within 30 min of procurement. Initially, the bovine tongue was washed several times using an ultrapure water and/or Hank’s Balanced Salt Solution (HBSS; Fisher Scientific, Loughborough, UK) to remove any impurities and lower any future microbial contamination. All experimental protocols adhered to Aston University practice for safe handling of animal tissues and cell culture experiments.

#### 2.1.1. Culture Media

The culture media was prepared in Iscove’s modified Dulbecco’s medium (IMDM, Gibco BRL, New York, NY, USA) containing 10% foetal bovine serum (FBS), 1:5 ratio of MCDB 153, and a triple cocktail of antibiotics (2.5 mL of 100 U/mL/100 μg/mL, penicillin/streptomycin; 2.5 mg/ mL gentamycin; and 0.5 mg/mL Amphotericin B).

#### 2.1.2. Isolation and Culturing of BTBCs

The fungiform papillae were carefully isolated using surgical blades and placed into small glass dish containing above stated culture media. Isolated tissue pieces were minced to smaller pieces using surgical blades and resulting cell suspension was then plated in 24-well plate Collagen I at 37 °C in humidified environment containing 5% CO_2_. Initially, the culture media was replaced after 48 h; and then, approximately twice a week when required. At the initial stage, the old media was carefully removed while leaving small amount (i.e., 1/3 of the old media) with the aim of minimising damage or lifting up of adhered cells by pipetting.

#### 2.1.3. Passaging and Propagation of Cultured Cells

Confluent BTBCs in individual wells were collected using cell scraper and seeded into T25 flask. However, if high cell density and higher number of wells with BTBCs were achieved, this step was skipped by directly culturing into a T75 flask. The propagation of T25 flask (or T75 flask) confluent BTBCs was further continued by harvesting the cells with a trypsin replacement, TripleTM Express (Fisher Scientific, Loughborough, UK), and seeding into several T75 flasks.

#### 2.1.4. Cryogenic Preservation and Thawing

Confluent BTBCs were removed using TripleTM EXpress and the cell suspension was centrifuged at 1000 rpm for 10 m. After discarding the supernatant, the cell pellet was re-suspended in Biofreeze media (VWR, Lutterworth, UK). The suspension of BTBCs was then aliquoted, as 1 mL suspension, into cryovials. Using an insulated container, the BTBCs containing cryovials were placed in −80 °C freezer for 2–3 days. The vials were then transferred into −196 °C liquid nitrogen.

To revive cryopreserved BTBCs, one of the banked cryovials was defrosted for 5 m at 37 °C in humidified environment containing 5% CO_2_. The contents of the vial were poured into T25 flask and approximately 10 mL media was added. The media was replaced after 48 h and then once in a week. Once confluent, the BTBCs were then transferred into T75 flask and maintained as routine.

### 2.2. Characterisation of BTBCs

#### 2.2.1. Expression of Taste Specific Genes

Taste bud cells (TBC) are broadly classified in to three subtypes: Type I, Type II (Bitter, sweet and umami) and Type III cells. Recent molecular studies have demonstrated the expression of several cell-type specific genes in each cell-type. Gene expression profile was investigated by designing primers against known taste specific genes, i.e., taste receptors and taste specific signal transduction pathways; briefly, *TRPM5* (bitter, sweet and umami), *T1R3* (sweet and umami), *ENTPD2* (Type I cells), *SNAP25* (Type III) and ten *T2Rs* (bitter).

#### 2.2.2. Primer Design and Specification

Primer pairs (listed in Supplementary Table S3) of genes listed in Supplementary Table S1, were designed with PrimerQuest software (Integrated DNA Technologies, Leuven, Belgium;accessed at —https://eu.idtdna.com and accessed: 18 July 2018 ) using common design parameters (as described in Supplementary Table S2). To avoid possible genomic DNA amplification, intron-spanning primers were designed where possible. The specificity of amplicons and primers was checked in silico using Primer-BLAST (National Centre for Biotechnology Information) alignment tool; accessed at: https://www.ncbi.nlm.nih.gov/tools/primer-blast/ and accessed: accessed date: 18 July 2018 [14].

#### 2.2.3. Total RNA Extraction

Population of BTBCs were harvested using above stated method and centrifuged at 400–800 g for 5 min. Total RNA was isolated using RNeasy Mini Kit (Qiagen, Hilden, Germany) following the supplier’s recommendation. To eliminate any traces of DNA, on column DNA digestion was performed using RNase-Free DNase Set (Qiagen, Hilden, Germany) where 80 µL of DNAse I working concentration was directly added to the spin column and incubated at room temperature 15–20 min.

Total RNA concentration and purity was checked using a Nanodrop^®^ ND-1000 (Thermo Scientific, Wilmington, MA, USA). A 260/280 ratio of ~2.0 was indicative of pure RNA (with minimal contaminants). Furthermore, RNA quality was assessed using non-denaturing 2% agarose gel electrophoresis.

#### 2.2.4. Reverse Transcription PCR (RT-PCR) and Electrophoresis

Total of 1 μg RNA was used for cDNA synthesis and PCR reaction in one step using the Qiagen One-Step RT-PCR Reaction Kit (Qiagen, Hilden, Germany). PCR amplification parameters were performed according to the manufacturer’s recommendations using MultiGene (Labnet International) in a final 50 µL reaction volume containing 0.6 μM of each primer; briefly, reverse transcription at 50 °C for 30 min, initial PCR activation step at 95 °C for 15 min, followed by 40 cycles of denaturation at 94 °C for 30 s, primer annealing at 55 °C for 30 s, and extension at 72 °C for 1 min and final extension at 72 °C for 10 min.

Following the PCR amplification, reaction mix was analysed by electrophoresis using non-denature agarose gel. The electrophoresis analysis was employed to ensure that only one PCR product with a correct amplicon size was produced at the end of reaction. Briefly, PCR products, alongside with DNA ladder (Thermo Fisher Scientific, Paisley, United Kingdom) were separated on 1.3% agarose gels stained with ethidium bromide for 2 h at 100 volts. Following electrophoresis, gels were visualised with the G-Box gel imaging system (Syngene, Cambridge, UK).

### 2.3. Immunocytochemistry

#### 2.3.1. Identification TRC Markers: α-Gustducin and PLCβ2

The protocol for identification of TRC markers α-gustducin and PLCβ2 adapted from Ozdener et al. [12]. The BTBCs were grown in coverslips in 6-well plate for approximately 3–4 days. After removing the culture media, the cells were washed twice with PBS. The BTBCs were then incubated in 4% paraformaldehyde (PFA) in PBS for 10 min. The PFA solution was replaced with blocking buffer for 40–60 min; the blocking buffer was prepared using 3% *v*/*v* normal goat serum, 3% *w*/*v* bovine serum albumin (BSA) and 0.3% *v*/*v* Triton x-100 in PBS. To achieve optimum staining concentration, primary antibodies, i.e., rabbit polyclonal Gαgust (I-20) and rabbit polyclonal PLCβ2 (Q-15) (Santa Cruz Biotechnology, Santa Cruz, CA, USA), were titrate in three serial dilutions, i.e., 1:100, 1:200 and 1:400, in the blocking buffer. The blocking buffer was removed and the cells with designated primary antibodies were incubated overnight at 4 °C. After overnight incubation with primary antibodies, cells were washed twice with PBS and then incubated with a goat anti-rabbit Alexa 488 (Fisher Scientific, Loughborough, UK) secondary antibody for 30 min at room temperature in the dark. The immunostaining experiments were performed with appropriate negative controls—including both primary- and secondary-only controls.

The cover slips were washed twice with PBS and ultrapure water; and mounted to microscope slides using Vectashield hardest mounting medium containing DAPI (4′,6-Diamidino-2-Phenylindole, Dihydrochloride) (Vector Laboratories, Burlingame, US). The slides were imaged on the same day after they dry using either Leica TCS SP5 II confocal imaging system or Leica Widefield Fluorescence Microscope (Leica Microsystems, Milton Keynes, UK).

#### 2.3.2. Identification of Bitter Membrane Receptors: T2R7

Receptors of the bitter membrane proteins (T2Rs) were also identified using the same immunocytochemistry procedure. Mouse anti-T2R7 (Santa Cruz Biotechnology, Santa Cruz, CA, USA), were prepared in three dilutions (i.e., 1:100, 1:200 and 1:400) and a goat Anti-Mouse Alexa Fluor 488 (Abcam) secondary antibody was added as above stated.

### 2.4. Flow Cytometry

TBCs express membrane proteins, e.g., taste specific receptors and ions channels, which have vital role in taste transduction. Flow cytometry-based analysis was performed using antibodies raised against the extracellular regions of these membrane proteins.

#### 2.4.1. General Flow-Cytometry Procedure

The flow cytometry protocol to identify and analyse cultured BTBCs was adapted from Menon et al. [15] and Sing et al. [16]. BTBCs were harvested using above stated enzymatic technique; and total cell number count and cell viability were determined. The BTBCs in suspension were centrifuged 400–800 g 4 °C for 5 min. The BTBCs cell pellet was re-suspended in ice cold wash buffer, i.e., MACS buffer, to an approximate cell density of 1 × 10^7^ cells/mL. MACS buffer was prepared using BSA (1%) and EDTA (2 mM) (Fisher Scientific) in PBS. After centrifugation, appropriate antibody concentration diluted in blocking buffer, i.e., 5% goat serum in PBS, was added and the cells were incubated for 15–30 min at 4 °C in the dark. The primary antibody was then removed and washed twice by centrifugation for 5 min using MACS buffer. All primary antibodies were prepared in three dilutions (i.e., 1:100, 1:200 and 1:400) unless specified. Appropriate fluorochrome-conjugated secondary antibodies, diluted in the blocking buffer, were added, and further incubated for 20–30 min at 4 °C in the dark. The BTBCs were washed twice with MACS buffer by centrifugation for 5 min. Cells were re-suspended in ice cold MACS buffer and cells were filtered through 85 µm mesh pluriStrainer filters prior flow cytometry analysis (pluriSelect Life Science, Leipzig, Germany). To identify and exclude dead cells, DAPI (0.02 μg/mL) was added just before flow cytometric analysis using MoFlo Astrios cell sorter (Beckman Coulter, High Wycombe, UK).

#### 2.4.2. CD73 Expressing Cells

CD73 is membrane protein reported to be specifically expressed in Type III cells [17]. Therefore, flow cytometry analysis was performed using PE conjugated anti-CD73 (Abcam) to identify BTBCs that express CD73. Antibody titration and optimisation was performed using three different concentrations, i.e., 1:100, 1:200 and 1:400. If no positively stained cells were observed, the specificity and suitability of the antibody was further studied using immunocytochemistry technique and confocal microscopy.

#### 2.4.3. TRPM5-Positive Cells

TRPM5 is specifically expressed in Type II cells (TRCs), i.e., cells sensitive to bitter, sweet and umami taste. Therefore, BTBCs were labelled using rabbit anti-TRPM5 primary antibody (Invitrogen, Thermo Fisher Scientific, Paisley, United Kingdom) and anti-rabbit Alexa Fluor 488 secondary antibody (Invitrogen). To find optimum labelling conditions, the applications of both primary and secondary antibodies were titrated using three dilutions (i.e., 1:100, 1:200 and 1:400) and two different concentrations, respectively.

#### 2.4.4. Bitter Receptors

There are more than 25 taste receptors, i.e., T2Rs, responsible for bitter taste transduction. BTBCs were stained with primary antibody raised against the extracellular regions T2R7 as stated above. Cells were labelled using three dilutions (i.e., 1:100, 1:200 and 1:400) of mouse anti-T2R7 (Santa Cruz Biotechnology, Santa Cruz, CA, USA) using and a secondary antibody goat anti-mouse Alexa Fluor 488 (Abcam, Cambridge, UK).

### 2.5. Calcium Imaging

TBCs release calcium ions from their internal stores to the cytosol following stimulation by taste compounds. This results in transient increase in intracellular free calcium ions which can be quantified using calcium imaging technique Fluo-4 AM. Calcium imaging experiment, as adapted from Ozdener et al. [12], was, accordingly, performed to investigate whether cultured BTBCs still retain their physiological functions of taste responsiveness post their isolation and cryopreservation. The dynamic calcium imaging experiments were conducted and analysed according to standard approaches used in our laboratory [18,19]. Cells were exposed for 50–100 ms depending on loading efficiency of particular culture, this was kept constant for each experiment. Data were analysed by extracting fluorescence values from regions of interest manually generated. Fluorescence values were normalised to baseline using excel to enable determination of mean agonist induced increases and statistical comparison between groups.

Briefly, BTBCs were grown on cover slips for approximately 3–4 days. The culture media was replaced with media containing Pluronic F127 (1% *w*/*v*) and Fluo-4 AM (10 µM) (Invitrogen). The cells were then incubated for 40–60 m at room temperature. Fluo-4 AM containing media was removed and replaced with normal cell culture media.

After 1-min perfusion with a culture media, 2 mM denatonium benzoate and 462.30 μM quinine hydrochloride in HBSS was perfused to the coverslips mounted on the calcium imaging chamber and images were taken every 5 s for 350 s. The results were then analysed using HCImage imaging software (Hamamatsu Photonics, Hamamatsu-city, Japan).

### 2.6. Preliminary Data Mining and Bioinformatics Studies on Bovine α-gustducin

#### 2.6.1. Chromosomal Mapping

Chromosomal mapping of the human and bovine bitter taste receptor (T2Rs) gene family and GNAT-3 was performed based on analysis of latest genomic information of both human (GRCh38.p13) and bovine genome assembly (ARS-UCD1.2/bosTau9). NCBI (https://www.ncbi.nlm.nih.gov/genome/gdv/?org=bos-taurus; accessed date: 16 May 2017), Ensembl (http://www.ensembl.org/Bos_taurus; accessed: 26 July 2020) and University of California Santa Cruz Genomics Institute (http://genome.ucsc.edu/; accessed: 26 July 2020) were used to collected genomic information and results from each database were cross examined.

#### 2.6.2. Protein Information

The National Centre for Biotechnology Information (NCBI) was used to data mine the accession number and analyse the gene location of bovine α-gustducin (i.e., G protein subunit alpha transducin 3 (GNAT-3)). The domains and functional sites of the protein were also identified using this database. The exons and gene-splicing variants, i.e., protein coding variants, were examined using the Ensemble database [20].

#### 2.6.3. Multiple Alignment Sequence and Matrix Identity

Initially, protein sequence alignments of bovine α-gustducin were performed using a web-based protein-protein basic local alignment search tool (BLASTP) [21]. The protein sequence alignment results of B.taurus, H.sapiens, R.norvegicus and M.musculus was obtained as a FASTA file from BLASTP and was uploaded to the web based Clustal Omega programme. Using multiple alignment sequences, amino acid sequences of orthologous proteins were compared and altered sequences were identified. NCBI’s Conserved Domain Database (CDD) [22] was used to further study if altered amino acid sequences rest in the functional sites and affect the function of the protein of interest. Furthermore, matrix identity percentage was determined between the orthologous proteins of four species using the Clustal Omega programme.

### 2.7. RT-qPCR Analysis of Taste Specific Genes

The expression of taste-specific genes in cultured BTBCs was analysed using RT-qPCR. In the initial characterisation, primer sets of eleven genes, including *GNAT-3*, *TRPM5* and *ITPR3* were designed, and the expression level of genes was quantified after normalisation with *PPIA*. Following the initial screening, forty-two genes were further studied using custom made RT-qPCR array. There was no need to include the genes which were studied in the initial screening in the array study.

#### 2.7.1. RT-qPCR Array

Custom made RT-qPCR array was designed using PrimePCR (Bio-Rad, Watford, UK) to quantify genes involve in taste transduction. The Kyoto Encyclopedia of Genes and Genomes (KEGG) pathway database (http://www.genome.jp/kegg/pathway.html; accessed: 20 December 2018) [23] was mainly used to identify potential target genes implicated in the taste transduction (map04742) (as shown in Supplementary Figure S3) and calcium signalling pathway (map04020) (as shown in Supplementary Figure S4); however, additional genes were also added from published articles.

The RT-qPCR array design was adapted from Amatori, Persico and Fanelli [24]. Quantification analyses of 42 bovine genes involved in taste transduction (full list of the genes with their Enseml ID, amplicon length and assay design can be found in Supplementary Table S4) were performed. Two reference genes *PPIA* and *RPS18* were also included in the qPCR array design. In addition, DNA contamination control assay (gDNA) was included to detect possible genomic contaminations; and positive PCR Control assay (PCR) and reverse transcription control assay (RT) were added as internal controls. To increase assay reliability and reproducibility, PCR array was designed to amplify each target gene in triplicates and qPCR assay was performed in four biological replicates of cultured BTBCs. PCR products of PCR array genes were evaluated using the melting curve analysis and agarose gel electrophoresis. Each cDNA was quantified using 2^−ΔCq^ method following normalisation using the reference gene *PPIA*.

#### 2.7.2. Reverse Transcription: First Strand cDNA Synthesis

First strand cDNA was synthesised using qScriptTM cDNA Synthesis Kit (Quanta Biosciences). Further, 1μg total RNA was reverse transcribed at a final 20 μL of reaction volume containing 4 μL qScript Reaction Mix, and 1 μL qScript Reverse Transcriptase using MultiGene thermal cycler (Labnet International). PCR amplification protocol was followed according to the manufacturer’s recommendations.

#### 2.7.3. Quantitative RT-PCR (RT-qPCR)

Real-time PCR analysis was performed on LightCycler^®^ 480 Instrument II (Roche, Burgess Hill, UK) using PowerUp™ SYBR™ Green Master Mix 2× (Applied Biosystems, CA, USA). Samples were investigated in triplicates; each well contained 10μL of final reaction volume with the following reagents: 5 μL PowerUp™ SYBR™ Green Master Mix, 0.5 μL forward and reverse primers (10 μM each; final concentration 0.5 μM each), 1 μL cDNA template (corresponding to final concentration of 50 ng) and 3.5 μL nuclease-free water. Thermal cycling parameters were performed according to the manufacturer’s recommendations.

## 3. Results

### 3.1. Bovine Taste Bud Cells (BTBCs): Isolation and In Vitro Culturing

The protocol for isolating and culturing of taste bud cells of bovine fungiform papillae were adapted from [12,25]. The fungiform papillae have an evident structure in the tip of the bovine tongue which was manipulated to specifically isolate the taste bud and minimise contamination from other cell types around the papillae.

Isolated taste buds placed in a small glass dish containing culture media were minced into small pieces using surgical blades. Taste buds have a garlic bulb-like structure [26] with very small opening to the epithelial surface via a taste pore [27]. Thus, mincing would enable the taste buds to open and allow the cells to grow individually attaching to the surface of the culture plate.

**Figure 2:**
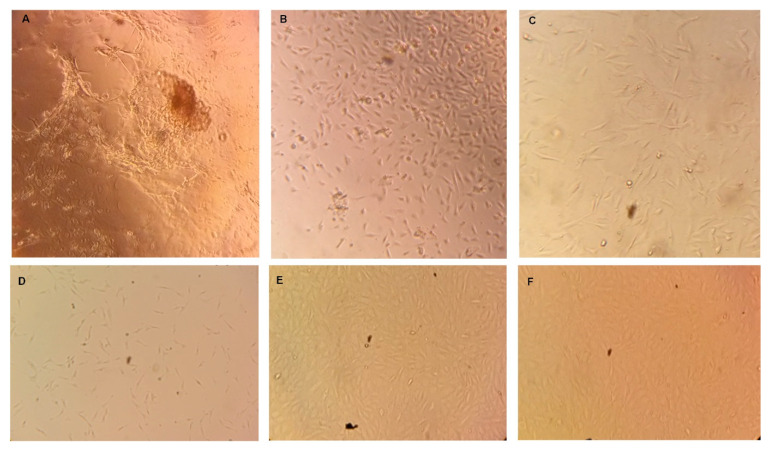
Isolation, culturing and reviving of bovine fungiform taste bud cells. (**A**) Taste bud cells in 24-well plates after 6 days of seeding. (**B**) Cells in 24-well plates after 9 days of seeding. (**C**) Cells in 25-mL flask after 48 hours of seeding. (**D**) Revived BTBCs following cryopreservation Passage-3 cells; (**E**) Passage-4 cells and (**F**) Passage-5 cells.

The tissue suspension was then plated in 24-well plate Collagen I at 37 °C in humidified environment containing 5% CO_2_. As shown below in Figure 2A–C, individual bovine cells and tissue portions started to adhere and grow in the cell culture plate. Initially, TBCs were heterogeneous with less degree of attachment. After six days, however (Figure 2A), proliferating cells were observed from underneath of bovine tongue cell clusters. These proliferating cells were well adhered to the culture plate and appeared to be more homogenous. After nine days of seeding (Figure 2B), non-adhered individual cells and tissue pieces were not observed in the culture plate.

Cultured cells reached confluency between 10–12 days of seeding and were then transferred to non-coated T25 cell culture flask. The BTBCs continued to adhere and grow in the culture flask; and had homogenous culture (Figure 2C). Cultured taste bud cells displayed morphological features consistent with taste cells in culture from other groups [12,13]. It is a well-known feature of cells in culture that they display altered morphology compared to in vivo [28,29]. Hence, Ozdener et al. [13] state that cultured human fungiform taste cells did not have the characteristic spindle-shaped morphology of in situ taste cells.

Batch variability and supply of cell line is one of the difficulties associated with primary cell line. To have continuous supply of stock cell lines, more than forty vials of passage-one and passage-two BTBCs were cryogenically preserved following successful isolation and culture. To ensure the cryogenically preserved BTBCs were still viable, some of the vials of BTBCs were revived and appeared to be morphologically identical (Figure 2D–F).

### 3.2. RT-PCR: Expression of Taste Specific Genes

Mature mammalian taste bud cells (TBC) are broadly classified in to three subtypes: Type I, Type II (bitter, sweet and umami) and Type III cells. One-step RT-PCR was employed to investigate expression profile of primary cultures of BTBCs. Total RNA was isolated using RNeasy Mini Kit according to the manufacturer’s recommendation. To eliminate any traces of DNA, on column DNA digestion was performed using RNase-Free DNase Set (Qiagen, Hilden, Germany). The purity and integrity of total RNA were then assessed using a Nanodrop^®^ ND-1000 (Supplementary Figure S2A) and were further analysed using gel electrophoresis (Supplementary Figure S2B). Furthermore, to avoid possible genomic DNA amplification, intron-spanning primers were designed where possible (Supplementary Table S3).

**Figure 3:**
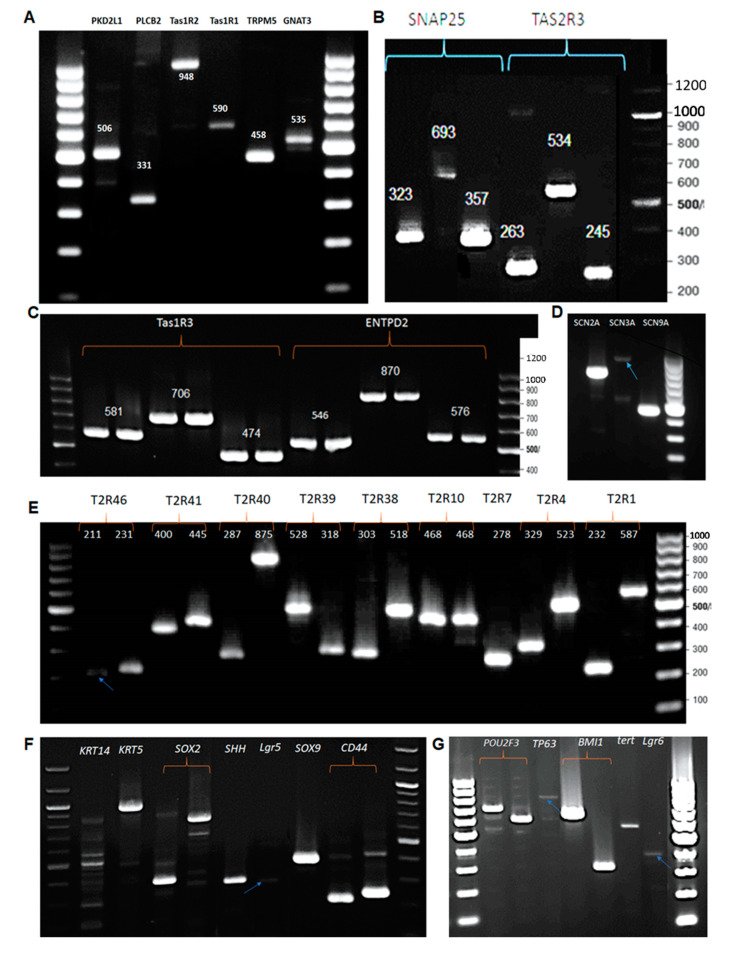
RT-PCR study of mRNA expression of taste cell markers including: (**A**) α-gustducin (*GNAT3*), *TRPM5*, *Tas1R1, Tas1R2, PLC-β2 and PKD2L1*; (**B**) *Tas2R3 and Snap25*; (**C**) *ENTPD2* and *T1R3*; and (**D**) voltage-gated sodium channels. (**E**) mRNA expression of bitter taste receptors; all the gene-specific primers yielded a single PCR product with a correct amplicon size except the second primer set of *T2R7*; and (**F**,**G**) cell fate and differentiation genes.

The expression of taste specific genes or genes involved in gustatory signalling pathways confirms the existence of mature taste bud cells. Most of the RT-PCR experiments were conducted using three different primers of each gene. *ENTPD2* is found to be clearly expressed as all three primers resulted in a single size with correct amplicon size (Figure 3C). The expression of *ENTPD2*, protein coding gene that hydrolyses ATP, clearly demonstrates the existence of Type I cell type in the mixed population of BTBCs. Similarly, single PCR product with expected amplicon size was detected in each of the three primers of *SNAP25* (Figure 3B) and the primer set of *PKD2L1* (Figure 3A). Type III cells of the TBCs express both *SNAP25* and *PKD2L1*

Several markers of Type II cells (bitter, sweet and umami) were also found to be expressed in the primary culture of BTBCs. These include α-gustducin (*GNAT3*), *PLC-β2*, *TRPM5*, encoding transient receptor potential M5, *T1R1*, *T1R2 T1R3, T2R3* and voltage-gated sodium channels (SCNs) (Figure 3A–D). Furthermore, several bitter taste receptors encoding genes, i.e., *T2R1*, *4*, *7*, *10*, *38*, *39*, *40*, *41* and *46*, were also found to be expressed (Figure 3E). These results suggest that expressed receptors (i.e., bitter –*T2Rs*, sweet—*T1R2*/*T1R3* and umami—*T1R1*/*T1R3*) and signalling compounds (i.e., α-gustducin (GNAT3), TRPM5 and voltage-gated sodium channels) may serve to eliciting Ca^2+^ response and cell depolarisation upon ligand (tastant) activation.

Several studies have demonstrated that progenitor/stem cells at the base (outside) of the taste bud and at the bottom of circumvallate and foliate trenches give rise to cells within taste buds [30,31,32]. Therefore, the next question was whether genes associated with cell fate and differentiation or progenitor cell markers were expressed in BTBCs.

Two keratinocytes, *KRT5* (cytokeratin 5) and *KRT14* (cytokeratin 14), were found to be expressed in BTBCs (Figure 3F). Additionally, the expression of several genes implicated as a taste bud cell stem or progenitor cell markers, i.e., *CD44*, *SOX9*, *SOX2*, *Lgr5*, *SHH*, *BMI1*, *TP63* and *POU2F3*, was detected in BTBCs (Figure 3F,G).

### 3.3. Immunocytochemistry

The main TRC markers, i.e., α-gustducin, PLC-β2 and membrane receptors, are involved in taste transduction mechanism of umami, sweet and bitter. Thus, the presence of these proteins confirms that the existence of the subpopulation of TBCs which are responsible for umami, sweet and bitter taste coding.

**Figure 4:**
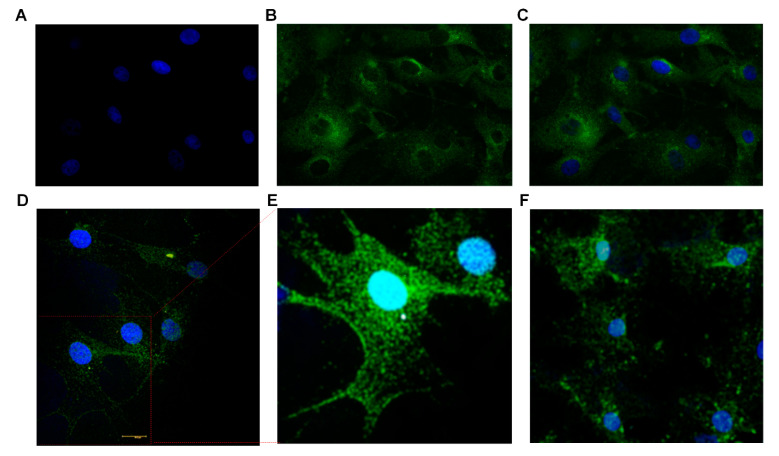
Immunocytochemistry studies demonstrating expression of taste-specific protein markers in cultured BTBCs. (**A–C**) Confocal images of cultured BTBCs expressing α-gustducin; (**A**) DAPI stained nucleus (blue), (**B**) Alexa Fluor 488 staining of the protein α-gustducin and (**C**) combined pictures. (**D**) PLCβ2 expressing cells and (**E**) the inset displays magnified view of the region on the red box; and (**F**) cells expressing bitter receptor T2R7.

Confocal images of BTBCs expressing the taste markers α-gustducin and PLCβ2 are shown below in Figure 4A–E. In Figure 4A–C, DAPI stained nucleus—large round (blue) (4A), Alexa Fluor 488 staining of the protein α-gustducin (4B) and combined pictures (4C) are presented. The immunostaining experiments were performed with appropriate controls (Supplementary Figure S1). Type II cells (taste receptor cells) are reported to have large round nuclei and while others (i.e., Type I and III) irregular elongated nuclei [33,34,35,36]. More importantly, Ma et al. stated that both α-gustducin and PLC-β2 expressing cells have large round nuclei which clearly supports the immunostaining findings [37]. The expression of the main TRC markers, i.e., α-gustducin and PLCβ2, were confirmed on the non-cryopreserved and revived cryopreserved cell lines; and this confirms that the cryopreservation of BTBCs did not affect the genotype of the TRCs which are responsible for the taste sensation of bitter, sweet and umami. Similarly, the expression of these proteins was also observed on both lower (Passage-2) and higher passages (Passage-5).

TRCs express families of G protein-coupled taste receptors (GPCRs) for sweet, umami and bitter tastants. As discussed above, more than 25 types of bitter receptors, i.e., T2Rs, have been identified in the human TRCs. The expression of T2R7 in cultured BTBCs is shown below in Figure 4F.

### 3.4. Flow Cytometry

Several studies have demonstrated TBCs express membrane proteins which have vital role in taste transduction, e.g., taste specific GPRCs and ions channels. Flow cytometry-based analysis was performed using antibodies raised against the extracellular regions of these membrane proteins.

**Figure 5:**
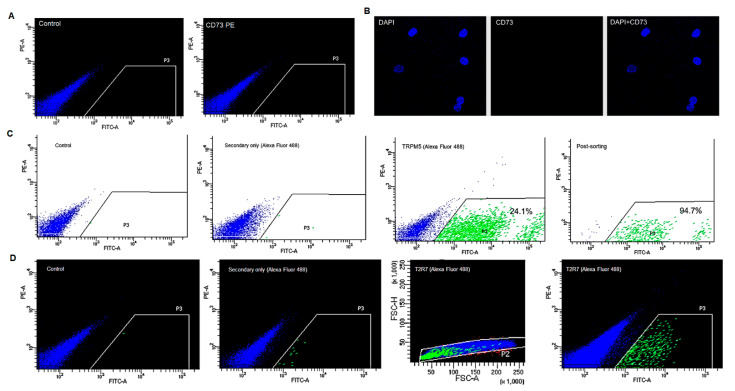
Flow cytometry analysis of cultured BTBCs. (**A**) BTBCs stained with anti-CD73 antibody; (**B**) confocal images to detect the expression of CD73 in BTBCs; DAPI stained nucleus (blue), protein of interest stained with anti-CD73 and combined pictures; (**C**) flow cytometry analysis of TRPM5-positive BTBCs showing negative control, secondary-only control, TRPM5-positive BTBCs (24.1%) and post-sorting purity analysis (94.7%); and (**D**) flow cytometry analysis of T2R7-positive BTBCs showing negative control, secondary-only control, excluded doublets shown as red dots outside the FSC-H vs. FSCA data plot and T2R7-positive BTBCs (0.4%).

Initial antibody titration and staining optimisation were performed on the antibody raised against CD73, a membrane protein reported to be expressed in Type III cells [17]. Three concentrations of the anti-CD73 antibody (i.e., 1:400, 1:200 and 1:100) were tested to investigate subpopulation of BTBCs expressing the CD73 membrane protein. Following flowcytometry analysis (Figure 5A), no expression of CD73 was observed in the cultured BTBCs of fungiform papillae. The expression of the CD73 protein was further explored using confocal microscopy. However, expression of CD73 was not detected in the cultured BTBCs replicating the flow cytometry results (Figure 5B).

Flow cytometry analysis of TRPM5 expressing BTBCs was performed following antibody titration and staining optimisation of anti-TRPM5 primary antibody and Alexa Fluor 488 secondary antibody. Approximately 24% subpopulation of BTBCs were found to express TRPM5. TRPM5-positive BTBCs (Figure 5C) were flow-sorted and post-sort analysis was performed to determine purity. Flow sorted TRPM5-positive BTBCs were not centrifuged, washed or stained for second round flowcytometry analysis. Flow sorted cells were rerun through flow cytometry and the purity was determined to be approximately 95%, (Figure 5C); a significantly high sorting purity [38]. Cell death and loss of fluorescence (particularly in dim population) during first round sorting may have contributed for the small loss in second round sorting of TRPM5-positive BTBCs.

The expression of a bitter taste receptor (T2R7) was also investigated using flowcytometry analysis using monoclonal anti-body raised against the extracellular regions of T2R7 and secondary antibody Alexa Fluor 488. The flow cytometry results (Figure 5D) demonstrated approximately 0.4% BTBCs were T2R7-positive cells confirming the presence of bitter sensitive sub population.

### 3.5. Calcium Imaging

Cultured BTBCs were also studied for functional responses. Cells grown in coverslips were initially loaded with Fluo-4 AM dye and then exposed to taste stimuli. The transient increase of the intracellular calcium level in response to taste stimuli was measured using change in Fluo-4 AM intensity.

**Figure 6:**
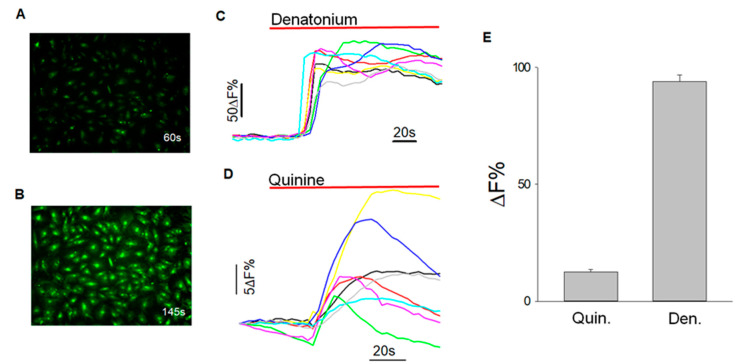
Calcium imaging to quantify tastant-evoked changes in intracellular calcium level. (**A**) BTBC before exposure to denatonium (2 mM) at 60 secs; (**B**) cells with change in fluorescence intensity after exposure to denatonium (2 mM) at 145 secs; (**C**) graph corresponding to response data of selected cells after exposure to denatonium benzoate (2 mM) after background subtraction; (**D**) graph corresponding to response data of selected cells after exposure to quinine HCl (462.30 μM); and (**E**) data are represented as mean (+/−SEM) of an increase in fluorescence (%) from baseline of three independent experiments. Denatonium-induced changes in relative fluorescence (%) was 93.97 +/−2.9 (data obtained from 187 cells of 3 experiments) and for quinine was 12.49 +/−1.11% (data obtained from 44 cells of 3 experiments).

BTBCs exposed to bitter stimuli denatonium (2 mM), and quinine (462.30 μM) exhibited an increase in fluorescent intensity (Figure 6A,B) and the change in fluorescent intensity was then quantified using HCImge software and data were represented as mean (+/−SEM) of an increase in fluorescence (%) from baseline of three independent experiments (Figure 6C,D). Denatonium-induced changes in relative fluorescence (%) was 93.97 +/−2.9 (data obtained from 187 cells of 3 experiments) and for quinine was 12.49 +/−1.11% (data obtained from 44 cells of 3 experiments) (Figure 6E). These results clearly demonstrate that the isolated BTBCs still retain their physiological functionality of taste transduction. These subpopulation of BTBCs were revived from the cryogenically preserved stock cells, and thus, these results also demonstrate that the cryopreservation did not affect the taste function of the cells.

### 3.6. Data Mining and Bioinformatics Studies on Bovine α-Gustducin

**Figure 7:**
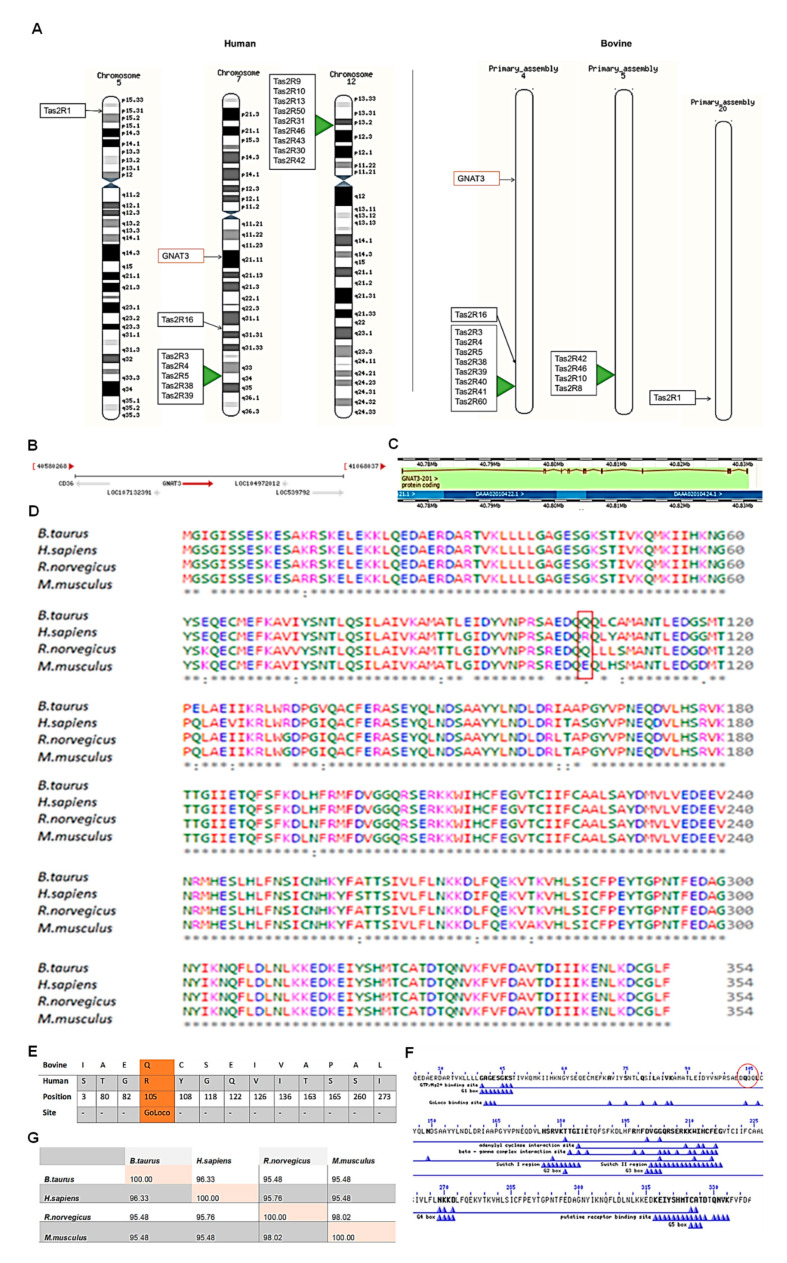
Preliminary data mining and bioinformatics studies on bovine α-gustducin. (**A**) Chromosomal mapping of the human and bovine type 2 taste receptor (T2R) gene family and *GNAT-3*. (**B**) Bovine *GNAT-3* gene location and neighbouring genes (i.e., *CD36*—receptor for fat); (**C**) Ensembl gene splicing image showing one protein coding variant of GNAT-3 gene and its associated exons—in filled rectangles and introns—as lines in between; (**D**) multiple alignment sequences of bovine GNAT-3 compared to human, mouse and rat GNAT-3 amino acid sequences; red box—mutation in the aspartate-glutamine-arginine triad (i.e., DQR); (**E**) altered amino acid residues found in the bovine protein compared to human GNAT-3 protein and associated functional site; (**F**) graphical summary of GNAT-3 conserved domains with its several functional features; red encircle amino acid sequences represents the DQQ <> DQR triad sequences; and (**G**) bovine GNAT-3 protein percent identity matrix compared to that of humans and rodent protein.

Chromosomal mapping of both human and bovine bitter receptor (Tas2Rs) gene family revealed that these genes exist predominantly within genomic clusters. Human T2Rs (Tas2Rs) are found clustered on chromosomes 7 and 12 and bovine T2Rs on chromosomes 4 and 5 in bovine (Figure 7A). Interestingly, α-gustducin encoding gene GNAT-3 is also located on the same chromosome, i.e., on chromosome 4 (human) and chromosome 7 (bovine).

Bovine GNAT-3 gene is found on chromosome 4 (between 40,775,885–40,830,619) as shown below in Figure 7B. The highlighted arrow (i.e., indicating to the right) shows that the GNAT-3 gene is located in the forward strand of the double helix DNA. The nucleotide sequence of the GNAT-3 gene has 15 exons and 14 introns (Figure 7C) with 1065 base pair (bp) nucleotides. However, GNAT-3 gene has only one protein coding variant.

The Clustal Omega (web based) programme was used to analyse multiple alignment sequences of GNAT-3 obtained from BLASTP results. Using multiple alignment sequences, the amino acid sequences of several proteins can be compared to the protein of interest and each other. The multiple alignment sequences of GNAT-3 are shown below in Figure 7D. The asterisk (*) indicates fully conserved residue, colon (:) greater than half and period (.) indicates weakly conservation similarity between groups (less than half). Overall, GNAT-3 is highly conserved in these species; and only a difference of thirteen amino acids were found between the bovine GNAT-3 protein and human GNAT-3.

The location of the amino acid sequences found altered between the bovine and human GNAT-3 were further analysed to identify whether any variation would affect the protein function (Figure 7E). One of the altered amino acid residues was found to lie in one functional site of the GNAT-3 protein, i.e., GoLoco binding site (Figure 7E,F). In the GNAT-3 protein, the aspartate-glutamine-arginine triad (i.e., DQR) is only conserved in the human protein sequence (Figure 7D). Thus, its role in taste transduction mechanism and inter-species difference should be studied further.

The pairwise identity scores matrix was generated using the Clustal Omega programme [39]. The rodents, i.e., rat and mouse, almost have an identical amino acid sequences with a 98.2% sequence match. Although similar percent of sequence similarity was observed between the human and bovine GNAT-3 compared to the rodents or vice-versa, the bovine and human GNAT-3 percentage similarity was slightly higher (Figure 7G).

### 3.7. RT-qPCR Analysis of Taste Specific Genes

**Figure 8:**
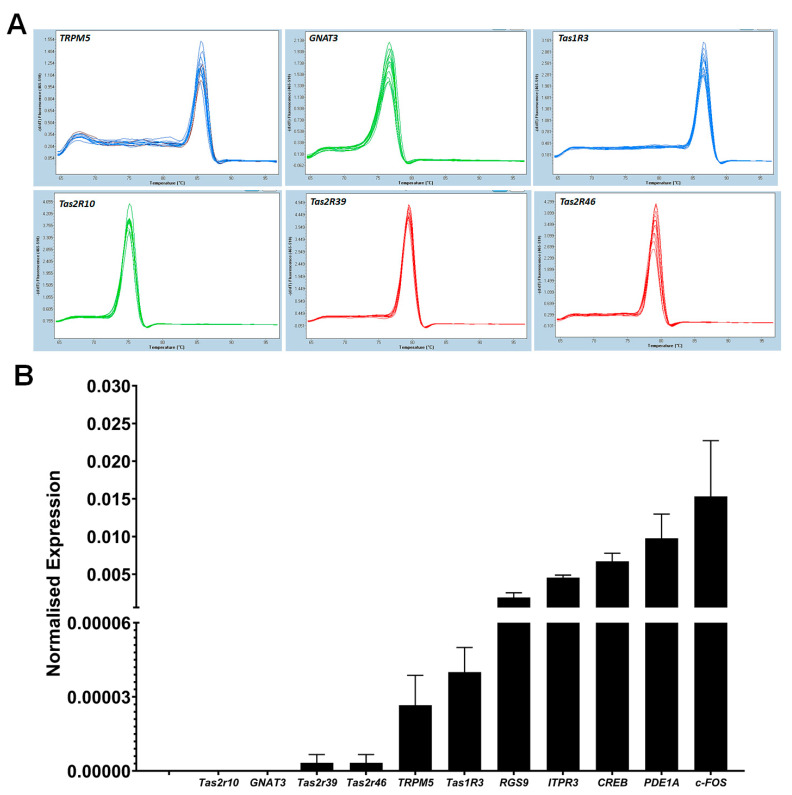
RT-qPCR analysis of taste specific genes; (**A**) melting curve analysis of taste specific genes; a single melting peak was observed for each target gene. The X-axis represents temperature (°C) and the Y-axis represents negative first derivative of fluorescence with respect to temperature (−dF/dT). (**B**) Quantification of taste gene mRNAs using RT-qPCR; each cDNA was normalised to the reference gene *PPIA* and normalised expression level (y-axis) of each gene was quantified using 2^−ΔCq^ method. Data are reported as mean of relative expression of three independent experiments and bars indicate standard error (±SE).

The expression of taste-specific genes in cultured BTBCs was analysed using RT-qPCR. In the initial characterisation, primer sets of eleven genes, including *GNAT-3*, *TRPM5 and ITPR3* were designed, and the expression level of genes was quantified after normalisation with *PPIA*. The qPCR assay was performed in three biological replicates of cultured BTBCs and each target gene was amplified in triplicate. PCR products of each genes were evaluated using melting curve analysis and a single melting peak was observed for the taste-specific genes (Figure 8A).

Initial screening of RT-qPCR study showed that taste specific genes were expressed in cultured BTBCs complementing the RT-PCR results. A single melting peak was observed for each target gene (Figure 8A). The expression level of these target genes was quantified using 2^−ΔCq^ method following normalisation with reference gene *PPIA.* RT-qPCR analysis demonstrated that all of these genes were lowly expressed in comparison to *PPIA* mRNA (Figure 8B). *Tas2r10, GNAT-3, Tas2R39 and Tas2R46* had the lowest mRNA abundancy. On the other hand, *CREB*, *PDE1A* and *c-FOS* had higher expression level corresponding ~200-, 100- and 67-fold lower than that of the reference gene.

Following the initial screening, forty-two genes were further studied using custom made RT-qPCR array. There was no need to include the genes which were studied in the initial screening in the array study.

### 3.8. RT-qPCR Array Quantification

Custom made RT-qPCR array was designed using PrimePCR (Bio-Rad, Watford, UK) to quantify genes involve in taste transduction. The Kyoto Encyclopedia of Genes and Genomes (KEGG) pathway database [23] was mainly used to identify potential target genes implicated in the taste transduction.

**Figure 9:**
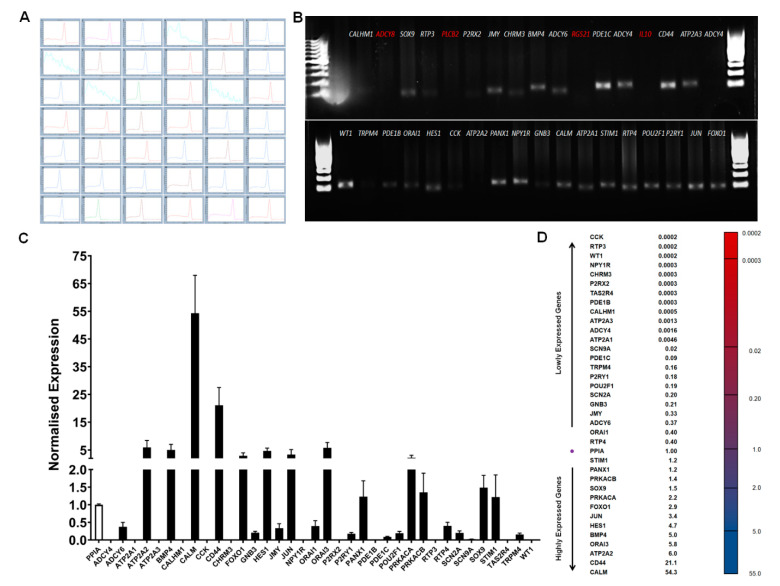
RT-qPCR array-based quantification of genes involved in taste transduction: (**A**) Melting curve analysis of 42 array genes; no melting peak was observed for the primer sets of *IL10* (4)*, RGS21* (7)*, PLCβ2* (14) *and ADCY8* (17). The X-axis represents temperature (°C) and the Y-axis represents negative first derivative of fluorescence with respect to temperature (−dF/dT). (**B**) Agarose gel analysis of 35 array genes following a qPCR reaction. A single PCR product with a correct amplicon size was observed for all genes except for the primer sets of *IL-10, RGS21, PLCβ2 and ADCY8*. (**C**) Quantification of taste gene mRNAs using custom made RT-qPCR array; each cDNA was normalised to the reference gene *PPIA* and normalised expression level (y-axis) of each gene was quantified using 2^−ΔCq^ method. Data reported as mean of relative expression of four independent experiments and bars indicate standard error (±SE). (**D**) Expression profile of RT-qPCR array genes arranged from lowly to highly expressed in BTBCs.

The qPCR assay was performed in four biological replicates of cultured BTBCs and each target gene was amplified in triplicate. Initially, PCR products of each array genes were evaluated using melting curve analysis and agarose gel electrophoresis. A single melting peak and a PCR product with a correct amplicon size were observed for all genes except for the primer sets of *IL-10, RGS21, PLCβ2 and ADCY8* (Figure 9A,B).

RT-qPCR screening of genes implicated in taste transduction pathway revealed that at least 38 genes of the 42 array genes are expressed in cultured BTBCs. The expression level of these target genes was quantified using 2^−ΔCq^ method following normalisation with reference gene *PPIA* (Figure 9C). Expression profile of RT-qPCR array data clearly show that the majority of these genes were lowly expressed in comparison to *PPIA* mRNA (Figure 9D). *CCK*, *RTP3* and *WT1* had the lowest mRNA abundancy almost 4000-fold lower than that of the reference gene. On the other hand, *FOXO1*, *c-JUN*, *HES1*, *BMP4*, *ORAI3* and ATP2A2 had expression level ranging three to six-fold. More interestingly, *CD44* and *CALM* genes were found highly expressed with normalised expression levels of 21.1 and 54.3, respectively.

## 4. Discussion

One of the primary limitations of cell-based taste assessment is the lack of a long-term supply of cultured TBCs. Thus, the aim of this study was to begin with isolating and long-term culturing bovine taste bud cells (BTBCs). Comparing to established mammalian TBCs isolation procedure, no enzymatic digestion during initial TBCs isolation and coverslip collagen coating were found necessary. Cryopreservation was performed to ensure continuous supply of stock cell lines. However, we have found that senescence starts to appear after Passage-7. Therefore, to overcome the difficulties associated with primary cell line, i.e., the batch variability and supply of cell line, work will commence on immortalising these cell lines.

Gene expression profiling using RT-PCR confirmed the expression of several mature mRNA transcripts of gustatory signal transduction and cell-type specific genes in BTBCs. The expression of *ENTPD2,* a protein coding gene that hydrolyses ATP, and *SNAP25,* synaptic protein coding gene, confirmed the existence of Type I and Type III cells, respectively. Both molecular and functionals have demonstrated that PKD2L1 is a candidate for sour taste in Type III cells [40,41]. Therefore, RT-PCR results of *PKD2L1* expression (Figure 3A) clearly indicate the presence of Type III cells in the cultured BTBCs. Furthermore, RT-PCR studies confirmed the expression of several genes associated with taste cell fate and differentiation or progenitor cell marker in BTBCs. Almost all these genes have been reported as stem cell or progenitor cell markers in several tissues [42,43,44].

Recent studies have started to scrutinise the role of several stem/progenitor cell marker genes on taste cell fate and differentiation. Cell lineage tracing studies of mice taste buds identified cells expressing *KRT5* and *KRT14* as the progenitor cells of TBCs both in the intragemmal basal and perigemmal cells [45,46]. Cells expressing *Sox2, Trp63, K14 and K5* have been identified as a bipotential precursor cells that can differentiate both into mature TBCs and adjacent epithelial cells [46]. Interestingly, *Lgr5*-positive cells were also found to co-express SOX2, KRT5 and KRT14 [35] as well as Sox9 and CD44 [47]. The homeodomain protein Pou2f3 (Skn-1a) has been found to be expressed and strictly required for the generation of Type II cells during cell type-specific differentiation of emerging TBCs [48]. *SHH* found to be expressed in the postmitotic basal cells may play a role in promoting taste cell differentiation [49].

Furthermore, several mRNA transcripts of gustatory signal transduction specific to type II cells (TRCs) were found to be expressed in the cultured BTBCs, i.e., *TRPM5, GNAT3, PLCβ2* and genes encoding voltage-gated sodium channels (SCNs). Expression of *T1Rs* and several *T2Rs*, bitter taste receptors encoding genes, established the presence of subset of BTBCs sensitive to sweet and umami (*T1Rs-positive)* and bitter (*T2Rs-positive*) taste, respectively. Complementing the RT-PCR results, both immunocytochemistry and flow cytometry studies have confirmed the expression of vital gustatory signal transduction proteins—α-gustducin, PLC-β2, TRPM5, T1R3 and a bitter receptor (T2r7). Several molecular and animal studies have demonstrated that ablation of taste specific proteins, i.e., α-gustducin and PLCβ2, significantly reduced or completely demolished sweet, amino acid, and bitter tastes [50,51,52,53,54].

In our flow cytometry studies, approximately 24% subpopulation of BTBCs were found to express TRPM5. Previous studies have also reported similar proportion of cells expressing TRPM5, i.e., 20–30%, and more importantly these cells were also found to co-express α-gustducin, PLC-β2 and IP3 receptor [55,56]. The flow cytometry results (Figure 5D) demonstrated ~0.4% of BTBCs were found to express the bitter receptor T2R7 confirming the presence of bitter sensitive sub population. Similarly, hT2R7 was found to be expressed in the lowest frequency in taste bud cells, only in ~0.7% taste cells [10]. Several candidate proteins for flow cytometry studies were selected, however, limited availability appropriate antibodies, particularly against extracellular regions of taste specific membrane proteins, hindered flow cytometry studies.

The expression of membrane protein of Type III cells, i.e., CD73, was not detect in BTBCs both through flow-cytometry and confocal imaging. Although, previously reported to be expressed in both the epithelium surrounding taste buds and Type III cells of mice circumvallate papillae, others [57], however, could not detect it on Type III cells rat circumvallate papillae. In this study, both flow-cytometry and immunocytochemistry findings affirm the later conclusion; therefore, expression of CD73 could be taste bud type- and/or species-specific.

Changes in intracellular level calcium level were quantified after BTBCs exposed to denatonium (2mM) and quinine (462.30 μM) exhibited increased fluorescence intensity confirming the presence of subset of cells responding to taste stimuli (i.e., at least to bitter taste stimuli).

Chromosomal mapping of both human and bovine bitter taste receptors and *GNAT-3* revealed that these genes exist within genomic clusters. More interestingly, further analysis using multiple sequence alignment and scoring matrix for pairwise sequence alignment has demonstrated that bovine α-gustducin protein has a higher sequence similarity to human α-gustducin comparing to that of rodents (i.e., rat and mouse).

The expression profile of the array genes may indicate their cellular function, hence highly expressed genes may be ubiquitous and/or are required for day-to-day maintenance while the lowly expressed have specific roles or only-expressed in specific cell-type [58]. Therefore, both highly expressed genes *CD44* and *CALM* (with normalised expression levels of 21.1 and 54.3, respectively) appears to be expressed in all subsets of TBCs and are required day-to-day maintenance. Calmodulin is a ubiquitous Ca^2+^ and sub-unit of several enzymes and channels [59]. CD44 plays an important role in various physiological events including in cell proliferation, cell-cell interaction, cell adhesion as well as cell migration [60]. Both bitter receptor T2r7 and cholecystokinin (CCK) encoding genes were of the lowly expressed genes (~4000-fold lower than the reference gene) and both are specifically expressed in bitter responding subset of TBCs [61,62]. The normalised expression level of *Tas2R10*, *-46* and *-4* was 2.2 × 10^−6^, 3.2 × 10^−6^, 4.6 × 10^−6^ (Figure 8B) and 3.0 × 10^−4^ (Figure 9D), respectively. Behrens et al. [10] investigated the expression level of three human bitter receptor genes and found that *TAS2R1*, *-16*, and *-38* standardised to reference gene GAPDH was ~1.5 × 10^−4^, 2.5 × 10^−5^ and 1 × 10^−4^. Our findings are consistent with this study (only available study, to our knowledge).

Therefore, combined results of RT-PCR, immunocytochemistry flow cytometry studies, and RT-qPCR analysis supplemented by calcium imaging, have proven that cultured BTBCs were TBCs which express taste-specific genes, taste specific protein markers and capable of responding to taste stimuli. Moreover, preliminary datamining and bioinformatics studies also demonstrated that the bovine α-gustducin have higher sequence similarity to the human orthologue comparing to that of rodents.

Given its direct clinical relevance and subsequent verbal communication of the taste quality, intensity and palatability, taste assessment using human taste panels still remains undisputed gold standard [63,64]. However, panel subjectivity, the running cost, low throughput, limited flexibility, potential toxicity and liability (e.g., assessing a new chemical entity) could make this assessment difficult to perform [65,66].

Animals, most commonly rodents, have also been used in conducting taste assessments. Given to the evident interspecies differences in taste sensitivity [67,68], ethical conditions in treatment of animals [69,70] qualitative only evaluation and low throughput make the use of animal taste assessments very limited [66].

Developing a high throughput cell-based taste assessment assay would overcome several hurdles associated with current taste assessment. Therefore, results from this work would pave a way to replace animal experimentation and provide surrogate cell-based throughput system to study human taste transduction. There are several advantages of utilising bovine tongue for isolation and culturing of primary taste bud cells. The bovine tongue is large in size and with higher numbers of taste buds. The fungiform taste buds on the bovine tongue are evident structure which facilitates to precisely isolate the taste bud. Most importantly, using bovine tongue is more cost-effective and ethically justifiable. As the bovine tongues used in this research were obtained from animals which were slaughtered to be consumed. In other studies, however, the animals, i.e., rodents and gorilla, were killed just to obtain very small number of the taste buds while the whole body is discarded.

In conclusion, the study has demonstrated isolation, optimisation of culturing conditions and novel characterisations of BTBCs including flowcytometry and RT-qPCR array. Presence of taste specific protein markers were qualitatively studied using immunostaining; however, the proportions of taste receptors cells were identified using quantitative flow cytometry analysis. Cultured taste cells might not be exact replicas of in-situ native cells. Culturing conditions could alter taste cells or taste stem cells, e.g., due to the lack of 3D anatomical and physiological interactions occurring in vivo. However, our findings show that cultured taste cells still provide a unique opportunity to study proliferation, differentiation, biochemical signalling pathways and genetic markers. Furthermore, in vitro taste assessment assays would enable the characterisation of intensity and specificity of known tastants, evaluation of new chemical entities, identification of molecules that alter or block taste transduction, and profiling of tastant-induced gene expression.

## Data Availability

All data generated and analysed during this study are included in this article and its supplementary information files.

## Supplementary Materials

The following are available online at https://www.mdpi.com/article/10.3390/cells10092285/s1, Figure S1: appropriate controls of immunostaining analysis: BTBCs stained only with DAPI (A); primary-only controls—stained with anti-α-gustducin antibody (B); and secondary-only control—stained with anti-rabbit Alexa 488 (C). Figure S2: RNA purity and integrity analysis: spectral pattern for RNA confirming a very characteristic profile of a typical nucleic acid (A) and two sharp bands representing 28S and 18S rRNAs (B). Figure S3: taste transduction pathway (map04742) from KEGG pathway database; and red arrows indicate genes included in the qPCR array study. Figure S4: calcium signalling pathway (map04020) from KEGG pathway database; and red arrows indicate genes included in the qPCR array study. Table S1: list of amplified genes, name and function in gustatory signal transduction. Table S2: parameters employed for primers design. Table S3: list of genes amplified, primers, and amplicon size. Table S4: list of RT-qPCR array genes, Ensembl ID, amplicon size and assay design (intron-exon).
