# Supplementary material for: Novel, Fully Characterised Bovine Taste Bud Cells of Fungiform Papillae
Source: Cells. 2021 Sep 2;10(9):2285. doi: 10.3390/cells10092285 (PMC8469975; doi:10.3390/cells10092285)
Supplement: Supplementary file 1 [file cells-10-02285-s001.zip › cells-1295596-supplementary.pdf]

Novel, fully characterised bovine taste bud cells of fungiform papillae

Habtom Ftuwi[1], Rhein Parri[1], Afzal R Mohammed[1,*]

[1]Aston Pharmacy School, Aston University, Birmingham, B4 7ET, U.K.

*corresponding author email: a.u.r.mohammed@aston.ac.uk

Supplementary information

I) Supplementary figures

Figure S1: Appropriate controls of immunostaining analysis: BTBCs stained only with DAPI (A); Primary-only controls – stained with anti-α-gustducin antibody (B); and secondary-only control – stained with anti-rabbit Alexa 488 (C)

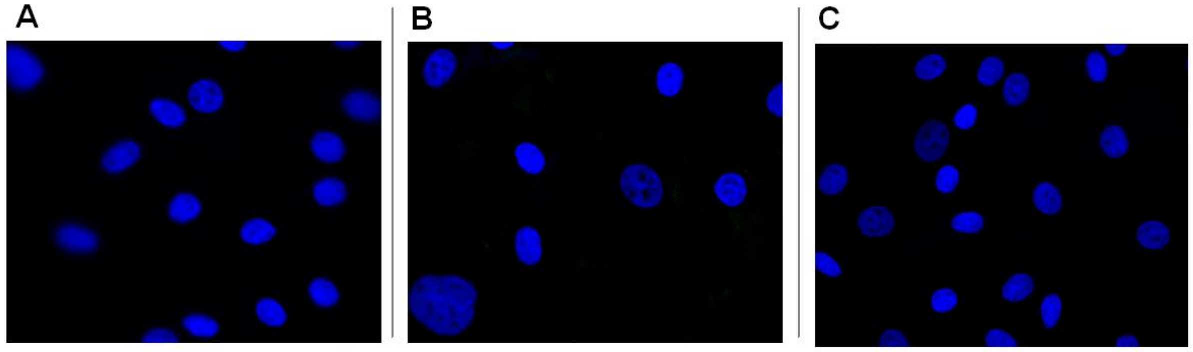

Figure S2: RNA purity and integrity analysis: spectral pattern for RNA confirming a very characteristic profile of a typical nucleic acid (**A**) and two sharp bands representing 28S and 18S rRNAs (**B**).

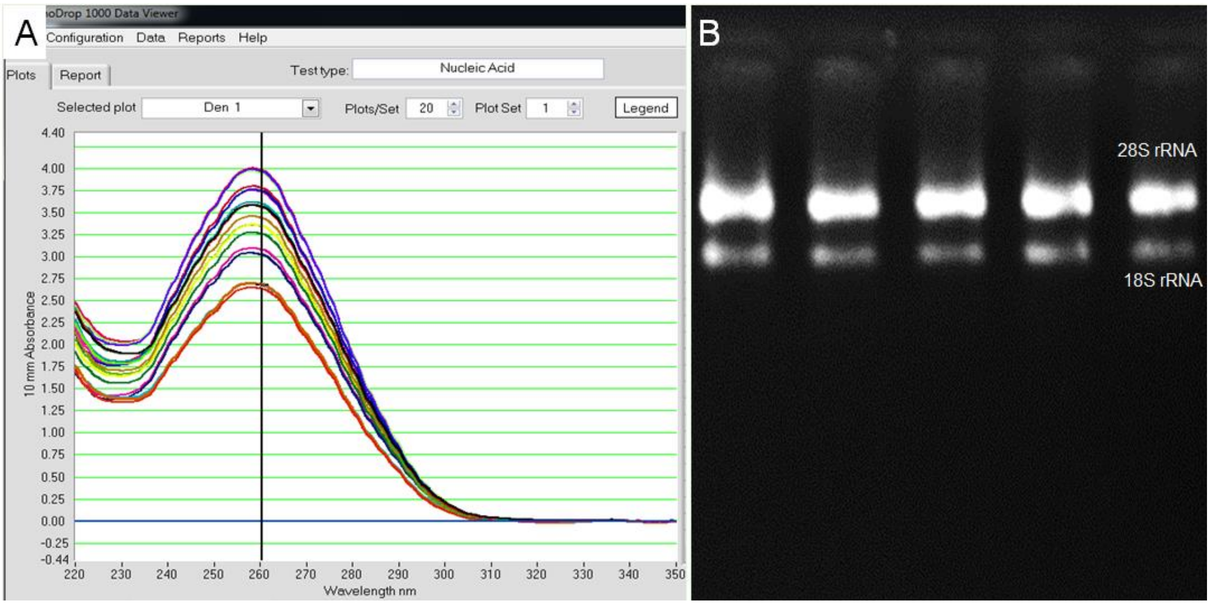

Figure S3: Taste transduction pathway (map04742) from KEGG pathway database; and red arrows indicate genes included in the qPCR array study.

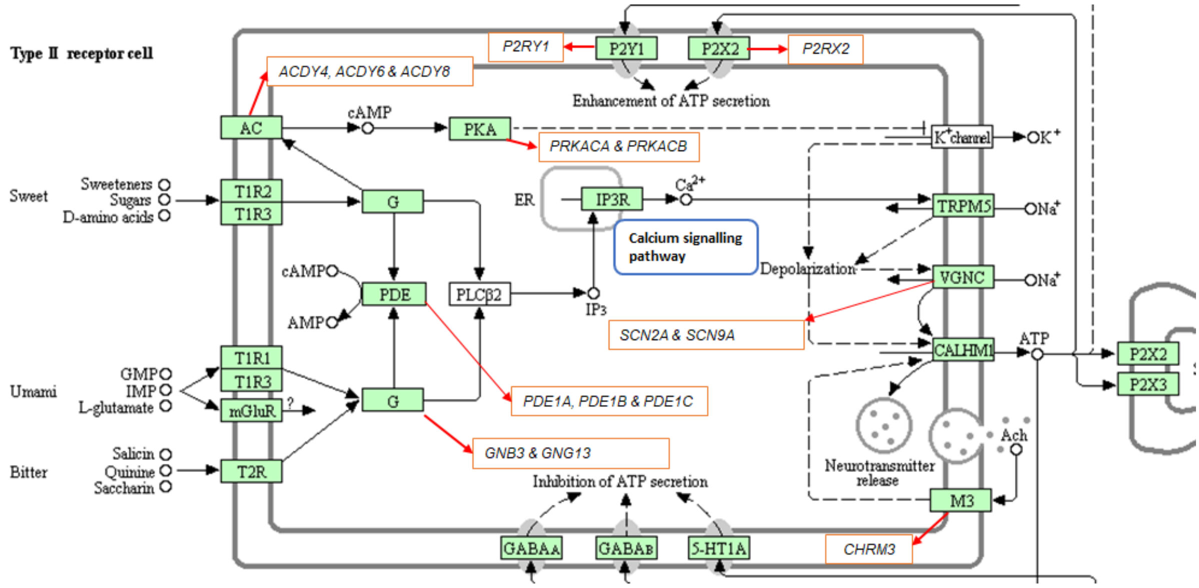

Figure S4: Calcium signalling pathway (map04020) from KEGG pathway database; and red arrows indicate genes included in the qPCR array study.

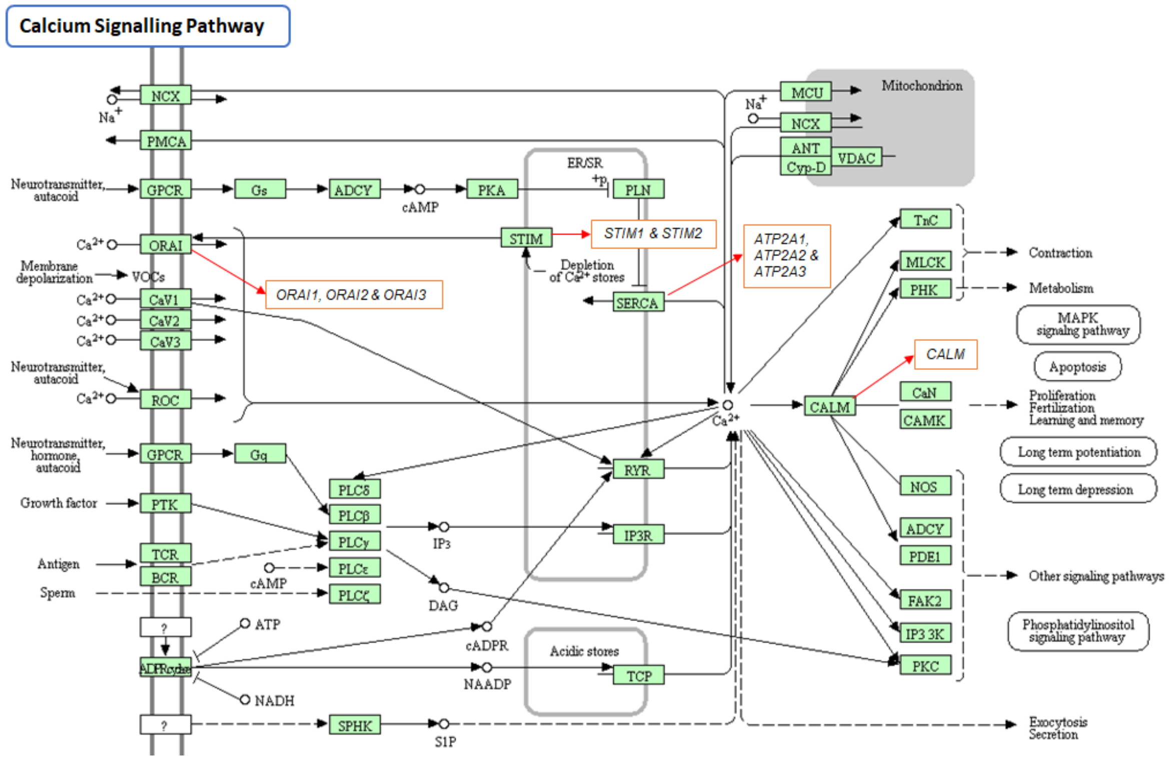

II) Supplementary tables

Table S1: List of amplified genes, name and function in gustatory signal transduction

Gene	Gene ID	Gene name	Cell pathway (function)
ENTPD2	NM_001105648.1	Ectonucleoside triphosphate diphosphohydrolase 1, NTPDase-2	Type I cells -ATPase
GNAT3	NM_001109982.1	Guanine nucleotide–binding protein, alpha transducing 3 (gustducin)	Type II - G protein
PLC-β2	NM_001191401.1	Phospholipase C Beta 2	Type II – taste transduction
TRPM5	XM_015461371.1	Transient receptor potential cation channel, subfamily M, member 5	Type II - Channel protein
SNAP25	NM_001076246.1	Synaptosomal-associated protein, 25 kDa	Type III - Synaptic protein
PKD2L1	XM_015469554.1	Polycystic kidney disease 2-like 1	Type III – sour receptor
TAS1R3	XM_015466888.1	Taste receptor, type 1, member 3	Type II - sweet and umami
TAS1R1	XM_002694166.4	Taste receptor, type 1, member 1	Type II – umami
TAS1R2	NM_001206529.2	Taste receptor, type 1, member 2	Type II - sweet
TAS2R3	NM_001046632.1	Taste receptor, type 2, member 3	Type II - Bitter receptor
TAS2R1	NM_001046636.1	Taste receptor, type 2, member 1	Type II - Bitter receptor
TAS2R4	NM_001046635.1	Taste receptor, type 2, member 4	Type II - Bitter receptor
TAS2R7	XM_002687793.3	Taste receptor, type 2, member 7	Type II - Bitter receptor
TAS2R7	XM_015463451.1	Taste receptor, type 2, member 7	Type II - Bitter receptor
TAS2R10	NM_001046626.1	Taste receptor, type 2, member 10	Type II - Bitter receptor
TAS2R38	XM_002687080.2	Taste receptor, type 2, member 38	Type II - Bitter receptor
TAS2R39	XM_609345.5	Taste receptor, type 2, member 39	Type II - Bitter receptor
TAS2R40	XM_010804639.2	Taste receptor, type 2, member 40	Type II - Bitter receptor
TAS2R41	XM_002687122.2	Taste receptor, type 2, member 41	Type II - Bitter receptor
TAS2R46	NM_001046629.1	Taste receptor, type 2, member 46	Type II - Bitter receptor
SCN2A	NM_001144109.2	Sodium Channel, Voltage-Gated, Type II, Alpha Subunit	Type II - Taste transduction
SCN3A	XM_002685332.4	Sodium Channel, Voltage-Gated, Type III, Alpha Subunit	Type II - Taste transduction
SCN9A	NM_001110787.2	Sodium Channel, Voltage-Gated, Type IX, Alpha Subunit	Type II - Taste transduction
CD44	NM_174013.3		progenitor cells
SOX9	XM_024981096.1	SRY (sex determining region Y)-box 9	progenitor cells
Lgr5	NM_001192520.3	Leucine-rich repeat containing GPCR 5	progenitor cells
Lgr6	XM_010813594.3	Leucine-rich repeat containing GPCR 6	progenitor cells
SHH	XM_024991301	Sonic hedgehog	progenitor cells

SOX2	NM_001105463.2	SRY (sex determining region Y)-box 2	progenitor cells
KRT5	NM_001008663.1	Keratin 5	progenitor cells
KRT14	NM_001166575.3	Keratin 14	progenitor cells
Tert	NM_001046242.1	Telomerase reverse transcriptase	progenitor cells
BMI1	NM_001038072.1	B lymphoma Mo-MLV insertion region 1 homolog	progenitor cells
TP63	NM_001191337.1	Tumour Protein P63	progenitor cells
POU2F3	NM_001205830.1	POU domain, class 2, transcription factor 3	progenitor cells

Table S2: Parameters employed for primers design

	**Min**	**Optimal**	**Max**
**Tm requirements (°C)**	59	62	65
**GC content (%)**	35	50	65
**Primer length (nt)**	17	22	30
**Amplicon size (bp)**	200		1000

Table S3: List of genes amplified, primers, and amplicon size

**Gene**	**Forward primer**	**Reverse primer**	**Length**	**Assay design**
ENTPD2	CCTGTGGCTGGGAAGTTTAT	TTGATCCTGGGCTGCATTAG	576	Intron-spanning (Exon 6-9)
	CGGCGGACAAAGAGAATGA	AGAAAGCAGAGAAGGCGATAAA	870	Intron-spanning (Exon 2-7)
	TCGCCTTCTCTGCTTTCTTC	CTGCATTAGGGACAGAGATGTT	546	Intron-spanning (Exon 7-9)
GNAT3	TCAACGACTCAGCAGCATAC	GCACAGGTCATGTGGGAATA	535	Intron-spanning (Exon 4-8)
PLCB2	CCCAGTACCAACTCCATCAATC	TGAGCTTCACAGCCTTCTTATC	331	Intron-spanning (Exon 22-32)
TRPM5	GTTGCGGCTGGAGAAGTATATC	GCTTCAGTTCGTCCAAGTAGTC	458	Intron-spanning (Exon 5-8)
TAS1R1	GGTTCAGCATCGAGGAGATAAA	GCCGGAGTAAACCACTACAA	590	Intron-spanning (Exon 2-3)
TAS1R2	CAGGAGGACTACAGCCAATATG	CCCTTGCTCGTTGAAGAAGA	948	Intron-spanning (Exon 2-4)

PKD2L1	CTTTCTCCTCGGCTTCTCTTAC	ATCTACACATCAGCGCATCTAC	506	
SNAP25	GTCAGGTGCTGTCTTTCCTT	AATCTGGCGGTTCTGTGTATC	693	Intron-spanning (Exon 7-8)
	GATACACAGAACCGCCAGATT	GAGCCACCAAATGACCACTAT	323	Exonic
	CAAATGCTGTTGGGCAAGATAG	GCAGTAGCTCTGTGGAATGT	357	Exonic
TAS1R3	ATCACCTGGGTCTCCTACAT	CAGGACAAGACAGAGCCTAAC	474	Exonic
	ATGCTGGCCTACTTCATCAC	ACCTCCCAATATCCCACTCT	706	Exonic
	GTGACCTCCAGAGTGCATAAG	GCAGGCAGTCTTGTATCTGT	581	Exonic
TAS2R3	TTTCCAGGGTGGTTGTATGG	CAGGGAGAGGATAAGCAGAAAG	245	Exonic
	AGTGTCTTCTACTGCCTGAAAG	GGAGTGGCCAGCAAGATATAA	534	Exonic
	GGTTTCATAGGGTGGGTCAAT	GCCACTTTCAGGCAGTAGAA	263	Exonic
TAS2R1	GTTTCCTGCTTGGCGATTTC	ATCAGGTAGTGGCAGAGATAGA	587	Exonic
	CATACAGCAGGGACACAAGAA	TTTCCAGGGTGGTTGTATGG	232	Exonic
TAS2R4	CCTTGCTCAACGTCTTGTATTG	GGAGGGTAAATGGTGGACATAA	523	Exonic
	CTTTCTGTCTTCACCACTCTCC	ACCAGGGTAGCAACTGAATATG	329	Exonic
TAS2R7	GTCAAGTGAAGGGCAGAGTATC	GGTTGGTTAGTGTCCAGAAGTAG	278	Exonic
TAS2R10	TGGCTGAAGGGTCACATTAC	TGAGTGTCCACAGGGATAGA	468	Exonic
	CACTAGCCTCAGCATCTTCTAC	TCAGGCACTGTCACACTTAAT	468	Exonic
TAS2R38	CTTCAGCTACCAGACCATCATC	CACAGAGTGACAGCACATACA	518	Exonic
	CAGTCCTGGAGTTTGCAGTAG	GGACAATCTTGGAGCAGTAGAG	303	Exonic
TAS2R39	CCATCAGAGCCATCAGCTATTT	GGGAAGAAGGAAGGTAGGAAGA	318	Exonic
	CTGGCTCAGTGTCTTCTACTTC	GGGTAGGTAGCCATGATGATTT	528	Exonic
TAS2R40	CTTGCCCTGTGAAGTCAGTAA	GAGAACCACGGGTGAGTAAAG	875	Exonic
	CCATCAGAGCCATCAGCTATTT	TCAAGGAGAGGAGAAGTCAGAG	287	Exonic
TAS2R41	CTCCTTCATTGTCACCCTACTG	GGTTGCCAAGGATGAGGATAA	445	Exonic
	GACCTGATCCTCTTTAGCTTGG	CCAGAATCCTGTTCCACTGATAG	400	Exonic
TAS2R46	CCTGTACGAAGGTCCACATAAA	GCCACAGAAACAACAGAAAGG	231	Exonic
	AGGAAACCTCACACCCTTTAC	AGGCCTCCAGATTGATATGATTAG	211	Exonic
SCN2A	GGGAGAGGGAGTAAAGGTAAGA	CAGCAGCAGAGGCAAGATAA	794	Exonic
SCN3A	GCCAATGTCCAGAAGGATACA	ATGTCCAGTAGGTGAGGTTAGA	977	Intron-spanning (Exon 8-11)
SCN9A	CTGGTGGTCCTCAACCTATTTC	CCCTTTGCTGTATTCGCTATCT	483	Intron-spanning (Exon 16-17)
CD44	AGCCAGAGAATACCTCGGATA	CACACCTTCTCCTACTGTTGAC	246	Intron-spanning (Exon 8-11)

	TCCAGCGAGCCAGAGAATA	GCAAACCGCGAGAATCAAAG	225	Intron-spanning (Exon 16-17)
SOX9	CGCAGATTCCCAAGACACTAA	GGCACGCTGTTCAAACTAAAG	402	Exonic
	GGAGGAAGTCGGTGAAGAAC	CATGTAGCTGAAGGTGGAGTAG	920	Intron-spanning (Exon 8-11)
Lgr5	CAGCCTCCGATCTCTGAATTT	GCCTTTACTCCACGGGTTAG	301	Intron-spanning (Exon 16-17)
SHH	TCACTCACTCACTCACACATAAC	CTCCTGTTAGCACTAGGTTTCC	351	Exonic
SOX2	CGCCCGCATGTACAACA	TGGAGTGGGAAGAAGAGGTAA	800	Exonic
	CCACCTACAGCATGTCCTATTC	GTTTCTTGCTGTCCTCCATTTC	369	Exonic
KRT5	TGGACTCAGAGCTCAGGAATA	TGTGACGACAGAGATGTTGAC	767	Intron-spanning (Exon 2-9)
KRT14	AAGAACCACGAGGAGGAAATG	TGGAGGATACATCTCTGGATGA	580	Intron-spanning (Exon 4-8)
Lgr6	TGTCAGAAGCTGGAGGAGAT	TCTTCCGGGTCCAGATCATAG	461	Intron-spanning (Exon 4-8)
Tert	GGCGTCAGACAACACTTAGA	GTGCAGGAAGAGGTTGAAGA	672	Intron-spanning (Exon 4-8)
BMI1	GGAGAAGTGGCTGATGAAGATAA	GCCATTCCTTCTCCAGGTATAAA	315	Intron-spanning (Exon 4-8)
	GCTCTAATGAAGACAGAGGAGAAG	CAGTCTCAGGTATCAACCAGATG	668	Intron-spanning (Exon 4-8)
TP63	CAGCCCATTGACTTGAACTTTG	ACTTGCCCATCTCTGGTTTC	794	Intron-spanning (Exon 4-8)
POU2F3	CCAGGTGGAGACAGGAAATG	GGGCCTCAAATCGTGAGATAG	632	Intron-spanning (Exon 4-8)
	GGGCTACCAGGATCTTCTTTAG	CAGGATGACGTTACTGTTCCA	689	Intron-spanning (Exon 4-8)

Table S4: List of RT-qPCR array genes, Ensembl ID, amplicon size and assay design (intron-exon)

Gene Name	Gene Symbol	RefSeq Accession No	Ensembl ID	Amplicon Length	Assay Design
Adenylate cyclase 4	*ADCY4*	NM_001099208	ENSBTAG00000018419	116	Intron-spanning
Adenylate cyclase 6	*ADCY6*	NM_001143877	ENSBTAG00000005464	78	Exonic
Adenylate cyclase 8	*ADCY8*	NM_001192841	ENSBTAG00000014600	97	Intron-spanning
ATPase, Ca++ transporting, cardiac muscle, fast twitch 1	*ATP2A1*	NM_001075767	ENSBTAG00000006541	103	Intron-spanning
ATPase, Ca++ transporting, cardiac muscle, slow twitch 2	*ATP2A2*	NM_001191430	ENSBTAG00000001398	112	Intron-spanning
ATPase, Ca++ transporting, ubiquitous	*ATP2A3*	NM_001114154	ENSBTAG00000014806	108	Exonic
Bone morphogenetic protein 4	*BMP4*	NM_001045877	ENSBTAG00000003835	110	Exonic
Calcium homeostasis modulator 1	*CALHM1*	XM_002698462	ENSBTAG00000007044	118	Exonic
Calmodulin 1 (phosphorylase kinase, delta)	*CALM*	NM_001242572.1	ENSBTAG00000025644	75	Exonic
Cholecystokinin	*CCK*	NM_001046603	ENSBTAG00000013027	120	Exonic
CD44 molecule	*CD44*	NM_174013	ENSBTAG00000011578	107	Intron-spanning
Cholinergic receptor, muscarinic 3	*CHRM3*	NM_174270	ENSBTAG00000008059	68	Exonic
Forkhead box protein O1	*FOXO1*	XM_002691748	ENSBTAG00000044105	88	Exonic
Guanine nucleotide binding protein (G protein), beta polypeptide 3	*GNB3*	NM_001192347	ENSBTAG00000016043	93	Exonic
Hairy and enhancer of split 1,	*HES1*	NM_001034678	ENSBTAG00000000569	76	Exonic
Interleukin 10	*IL10*	NM_174088	ENSBTAG00000006685	120	Intron-spanning
Junction mediating and regulatory protein, p53 cofactor	*JMY*	XM_002690458	ENSBTAG00000025856	92	Exonic
Jun proto-oncogene (JUN)	*JUN*	NM_001077827	ENSBTAG00000004037	95	Exonic
Neuropeptide Y receptor Y1	*NPY1R*	NM_001045898	ENSBTAG00000047586	94	Exonic
ORAI calcium release-activated calcium modulator 1	*ORAI1*	NM_001099002	ENSBTAG00000004457	67	Exonic
ORAI calcium release-activated calcium modulator 3	*ORAI3*	NM_001193202	ENSBTAG00000002344	115	Exonic
Purinergic receptor P2X, ligand-gated ion channel, 2	*P2RX2*	NM_001192643	ENSBTAG00000007496	61	Exonic
Purinergic receptor P2Y, G-protein coupled, 1	*P2RY1*	NM_174410	ENSBTAG00000001465	98	Exonic
Pannexin 1	*PANX1*	NM_001245925	ENSBTAG00000012010	119	Intron-spanning
Phosphodiesterase 1B, calmodulin-dependent	*PDE1B*	NM_174415	ENSBTAG00000004337	101	Intron-spanning
Phosphodiesterase 1C, calmodulin-dependent 70kDa	*PDE1C*	XM_002686835	ENSBTAG00000002739	111	Intron-spanning
Phospholipase C, beta 2	*PLCB2*	NM_001191401	ENSBTAG00000019079	93	Intron-spanning
Bos taurus POU class 2 homeobox 1	*POU2F1*	NM_001075582	ENSBTAG00000024534	100	Intron-spanning
Peptidylprolyl isomerase A (cyclophilin A)	*PPIA*	NM_178320	ENSBTAG00000012003	89	Exonic
Protein kinase, cAMP-dependent, catalytic, alpha	*PRKACA*	NM_174584	ENSBTAG00000006642	79	Intron-spanning
Protein kinase, cAMP-dependent, catalytic, beta	*PRKACB*	NM_174585	ENSBTAG00000011953	73	Exonic
Regulator of G-protein signaling 21	*RGS21*	XM_002693930	ENSBTAG00000037494	93	Intron-spanning
Ribosomal protein S18	*RPS18*	NM_001033614	ENSBTAG00000002648	117	Intron-spanning
Receptor (chemosensory) transporter protein 3	*RTP3*	XM_010817952	ENSBTAG00000018767	80	Exonic
Receptor (chemosensory) transporter protein 4	*RTP4*	NM_001075961	ENSBTAG00000032265	105	Exonic
Sodium channel, voltage-gated, type II, alpha subunit	*SCN2A*	NM_001144109	ENSBTAG00000038180	84	Intron-spanning
Sodium channel, voltage-gated, type IX, alpha subunit	*SCN9A*	NM_001110787	ENSBTAG00000002425	117	Intron-spanning
SRY (sex determining region Y)-box 9	*SOX9*	XM_005221337	ENSBTAG00000045824	85	Exonic
Stromal interaction molecule 1	*STIM1*	NM_001035409	ENSBTAG00000013109	85	Intron-spanning
Taste receptor, type 2, member 4	*TAS2R4*	NM_001046635	ENSBTAG00000018440	109	Exonic
Transient receptor potential cation channel, subfamily M, member 4	*TRPM4*	XM_002695205	ENSBTAG00000006139	115	Exonic
Wilms tumor 1	*WT1*	XM_003587002	ENSBTAG00000047268	110	Intron-spanning
